# Effect of Korean red ginseng on cold hypersensitivity in the hands and feet: study protocol for a randomized controlled trial

**DOI:** 10.1186/1745-6215-14-438

**Published:** 2013-12-19

**Authors:** Kyoung-Sun Park, Jin-Woo Kim, Jun-Young Jo, Deok-Sang Hwang, Chang-Hoon Lee, Jun-Bock Jang, Kyung-Sub Lee, Inkwon Yeo, Jin-Moo Lee

**Affiliations:** 1 Department of Gynecology, College of Korean Medicine, Kyung Hee University, Hoegi-dong, Dongdaemun-gu, Seoul 130-701, Republic of Korea; 2 Department of Statistics, College of Science, Sookmyung Women’s University, Cheongpa 2 ga, Yongsan-gu, Seoul 140-742, Republic of Korea

**Keywords:** Cold hypersensitivity in the hands and feet, Korean red ginseng, Infrared thermography, Cold stress test, Distal-dorsal difference

## Abstract

**Background:**

Cold hypersensitivity in the hands and feet (CHHF) is one of the most common complaints among Asians, especially in women. Korean red ginseng (KRG), which is a steamed form of *Panax ginseng,* has vasodilating action in the peripheral vessels and increases blood flow under cold stress. However, few studies have evaluated the effect of KRG on cold hypersensitivity.

**Methods/Design:**

This trial is a randomized, double-blind, placebo-controlled trial in 80 CHHF patients. The trial will be implemented at Kyung Hee University Hospital at Gangdong in Seoul, Korea. The participants will take KRG or a placebo for eight weeks, after which they will be followed-up for four weeks. During the administration period, six capsules of 500 mg KRG or placebo will be provided twice a day. The primary outcome is change of skin temperature in the hands between baseline and after treatment. The secondary outcomes include the visual analogue scale scores of cold hypersensitivity in the hands, change of skin temperature and the VAS scores of cold hypersensitivity in the feet, the recovery rate of the skin temperature by the cold stress test of the hands, the distal-dorsal difference of the hands, power variables of heart rate variability, and the 36-item short form health survey.

**Discussion:**

This study is the first trial to evaluate the efficacy of KRG on CHHF by using infrared thermography. Our study will provide basic evidence regarding CHHF.

**Trial registration:**

CliniacalTrials.gov NCT01664156

## Background

Generally, cold hypersensitivity refers to the condition in which patients feel excessively cold at low temperatures, and cold hypersensitivity in the hands and feet (CHHF) is one of the most common complaints among Asians, especially in women. According to a previous study on the genetic etiology of CHHF, the ratio of CHHF among females to males was found to be approximately 3:2 [1]. In particular, the prevalence of CHHF in Japanese, Korean and Chinese women is 54.3%, 25% and 20%, respectively [2]. CHHF can lower the quality of life by interfering with daily activities and, according to Korean medicine, may also be related to gynecological disorders such as menstrual pain, irregular bleeding, leucorrhea and infertility [3]. The known causes of CHHF are autonomic nervous dysfunction, peripheral neuropathy, median neuritis, anemia and diabetes mellitus [4]. However, the pathophysiology of CHHF remains unknown and a uniform treatment has not been established.

The root of *Panax ginseng* C.A. Meyer has been used worldwide for thousands of years for treatment of diverse diseases including diabetes [5], postmenopausal symptoms [6] and cancers [7]. Korean red ginseng (KRG) is a steamed form of *Panax ginseng*, which suggests that chemical transformation of active physiological properties occur involving ginsenosides, polysaccharides, peptides and polyacetylenic alcohols [8]. As a consequence of these biochemical changes, KRG acquires additional physiological activities. Steaming ginseng at temperatures over 100°C, results in the production of the gisenosides Rg3 and Rg5, which are not present in raw ginseng. Moreover, steamed ginseng is more potent at inducing endothelium-dependent vasodilation and radical-scavenging [9].

Many studies have suggested that KRG benefits vascular functions. KRG and its ginsenosides had vasodilating action on peripheral vessels and increased the blood flow under cold stress in animal experiments [10]. The ginsenosides isolated from KRG have been reported to cause vasodilation in an endothelium-dependent and nitric oxide- (NO-) mediated manner in a rabbit model [11]. Especially, the ginsenoside Rg3 was proven to inhibit vascular contraction as a consequence of NO production in a rat *in vitro* and *in vivo*[12]. Besides, in humans, KRG improved the vascular endothelial dysfunction in patients with hypertension through increasing synthesis of NO [13]. In addition, according to a randomized, controlled, double-blind trial, KRG was proven to improve arterial stiffness as measured by augmentation index [14]. According to Korean medicine, KRG is considered to have a warm quality and is used as a tonic to invigorate the body and enhance the ‘*Qi*’, which subsequently improves blood flow [15].

The vasodilating action and the warm quality of KRG provide a basis for its potential efficacy on CHHF. However, only one clinical trial has evaluated the effect of KRG on CHHF [16]. In the previous trial, the participants were divided into three groups that received KRG, placebo or nifedipine (a vasodilator). The participants then submerged their hands in cold water and the time until the pain became intolerable was measured. The tolerance time to cold stress significantly increased after administration of KRG compared to the placebo and nifedipine. However, the measurement of the tolerance time to cold stress used in the previous study is a subjective assessment tool.

The aim of the current study is to evaluate the efficacy of KRG on CHHF by using an objective assessment tool, infrared thermography. The hypothesis is that KRG will reduce CHHF more effectively than placebo after eight weeks of administering either KRG or placebo. The outcome measures include changes of skin temperature and the visual analogue scale (VAS) scores of CHHF, the recovery rate (RR) of skin temperature by the cold stress test (CST) of the hands, the distal-dorsal difference (DDD) of the hands, power variables of heart rate variability (HRV), and the 36-item short form health survey (SF-36).

## Methods/Design

### Trial design

**Figure 1:**
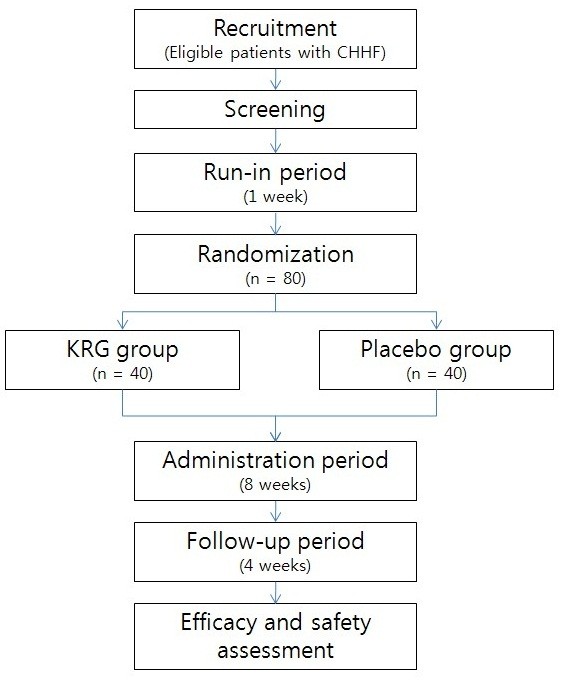
**Study flow chart.** CHHF, cold hypersensitivity in the hands and feet; KRG, Korean red ginseng.

This trial is a randomized, double-blind, placebo-controlled trial in 80 CHHF patients. The trial will be implemented at Kyung Hee University Hospital at Gangdong in Seoul, Korea. The participants will take either KRG or placebo for eight weeks, after which they will be followed-up for four weeks. During the administration period, six capsules of 500 mg KRG or placebo will be provided twice a day (one hour after breakfast and dinner). A flow chart of the entire trial is shown in Figure 1.

### Participants

#### Inclusion criteria

The patients who meet all threeof the following inclusion criteria items will be included:

(1) Females aged 16 to 60 years

(2) Females complaining of CHHF

(3) A thermal difference greater than 0.3°C between the palm and the upper arm

#### Exclusion criteria

Patients who report the following conditions will be excluded:

(1) Skin ailments, radiculopathy, thrombophlebitis and injuries affecting infrared imaging

(2) Alcohol abuse or alcoholism

(3) A history of cancer within the past five years

(4) Severe depression or mental illness

(5) Taking hypertensive or diabetic medications or thrombolytic agents

(6) Pregnant or breastfeeding

(7) Severe heart, pulmonary, hepatic and renal diseases (assessed by self-reporting and a blood test at the screening visit)

(8) Allergies to KRG or ginseng

(9) Ingestion of herbal medicine or nutritional supplements within a week before participation

(10) Participation in another clinical trial within the past three months

### Interventions

The KRG (Korean Red Ginseng powder capsule®) was manufactured by Korea Ginseng Corporation (Seoul, Korea) from the root of six-year-old *Panax ginseng* C.A. Meyer (family Araliaceae) harvested in Korea. The raw ginseng was cultivated and managed by Good Agricultural Practices established by the Rural Development Administration. The manufacture of KRG was processed according to Korean Good Manufacturing Practices and permitted and regulated by the Korean Food and Drug Administration. KRG was made by steaming fresh ginseng at 90 to 100°C for three hours, followed by drying at 50 to 80°C. KRG capsules containing KRG powder were prepared from ground red ginseng (KRG capsule, 500 mg/capsule). A total of 3 g of KRG powder contained 2 g of carbohydrates and 1 g of protein. The capsule was made of hydroxypropyl methylcellulose, pectin, purified water, sucrose fatty acid ester, glycerin, calcium gluconate and glacial acetic acid. KRG was analyzed by high-performance liquid chromatography. KRG contained the following active compounds: -Rb1, 5.61 mg/g; -Rb2, 2.03 mg/g; -Rc, 2.20 mg/g; -Rd, 0.39 mg/g; -Re, 1.88 mg/g; -Rf, 0.89 mg/g; -Rg1, 3.06 mg/g; -Rg2(s), 0.15 mg/g; -Rg3(r), 0.08 mg/g; -Rg3(s), 0.17 mg/g; -Rh1, 0.30 mg/g, and other minor ginsenosides. The standard chemical components in KRG were the total amount of Rb1 and Rg1 (6 mg/g). The examination of the remaining agricultural chemical was also conducted. The tolerance limit for pesticide residue of KRG was 0.5 mg/kg according to the regulation of Korean Food and Drug Administration. The voucher specimens were kept at the laboratory of Korea Ginseng Corporation (Seoul, Korea). The lot number of KRG that will be used in our trial is 30032005.

The participants assigned to the KRG group will be required to take six capsules of 500 mg KRG twice a day (one hour after breakfast and dinner), accounting for a total of 6 g in a day. The dosage of KRG was determined based on the total amount of Rb1 and Rg1, according to Korean Food Standards Codex. The participants assigned to the placebo group will take placebo capsules similar to the color, flavor and scent of KRG. Placebo was made of cornstarch, natural coloring (Brown CG-11771, JEY’s F.I. Inc., Sungnam Korea), brown caramel coloring (Bolak Co., Hwasong Korea) and red ginseng flavor (C80509, French Korean Aromatics Co., Yongin Korea). The lot number of placebo that will be used in our trial is 30032006. Samples of the placebo were also kept at the Korea Ginseng Corporation (Seoul, Korea). After several attempts, a placebo that could not be distinguished from the real KRG by 20 healthy persons was successfully produced. Besides, at the end of the study, all participants will be asked whether the experimental agents that they had taken were real or placebo in order to evaluate the success of blinding. In addition, the patients will be given a diary, which they will be asked to fill in twice a day, after taking KRG or placebo. The diary will be used to check their compliance to the administration. The patients’ data will be excluded from the per-protocol analysis unless their compliance rate is more than 70%.

### Objectives

The objectives of the current study are (1) to evaluate the efficacy of KRG on CHHF and (2) to establish basic evidence via the evaluation of CHHF by using objective assessment tools.

### Outcomes

#### Primary outcome

The primary outcome is a change of skin temperature in the hands between baseline (visit 2) and after treatment (visit 4). The skin temperature will be obtained by infrared thermography. For the infrared thermography examination, the patient will be asked to avoid hot showers, hot packs, smoking, exercise, acupuncture and stimulants such as caffeine for two hours before the examination. The patient will be acclimatized to room temperature (25 ± 1°C) for 15 minutes and seated comfortably on a chair without physiological or psychological stress. Subsequently, the patient will stand in an anatomical posture. Thermal images of the palm and the upper arm will be obtained to assess cold hypersensitivity in the hands. The difference between the palm and the upper arm, which is related to the severity of cold hypersensitivity in the hands, will then be calculated.

#### Secondary outcome

The secondary outcomes include the VAS scores of cold hypersensitivity in the hands, change of skin temperature and the VAS scores of cold hypersensitivity in the feet, RR of the skin temperature by the CST of the hands, the DDD of the hands, power variables of HRV, and the SF-36 between the baseline (visit 2) and after the treatment (visit 4). A 100-mm VAS measurement will be used to assess the severity of CHHF. The VAS scores will be rounded to the nearest integer in millimeters.

The CST is used to examine the function of recovery after cold stress. The patient will be seated and the baseline thermal images of the dorsum of both hands will be acquired with infrared thermography. Subsequently, both hands will be submerged up to the wrist in cold water (20°C) for a period of one minute. The hands will then be taken out of the cold water and will be carefully wiped and dried with a dry towel. Thermal images of the dorsum of both hands will be obtained immediately after immersion and six minutes later. The RR of the skin temperature at six minutes will be calculated as follows:

$$\mathrm{RR}=\left(T_{6}-T_{0}\right)/\left(T_{\mathrm{base}}-T_{0}\right)\times 100\left(\%\right)$$

T_base_: baseline skin temperature

T_0_: skin temperature immediately after cold stress

T_6_: skin temperature six minutes after cold stress

Recently, the thermographic DDD has been used as a parameter to evaluate the severity of vasoconstriction [17,18]. The DDD was calculated by subtracting the temperature of the finger from that of the dorsum [19]. Therefore, the DDD is considered positive if the finger is colder than the dorsum, and a high DDD indicates severe CHHF.

HRV is used to study the activity of the autonomic nervous system indirectly. Low-frequency (LF) power is thought to be strongly related to sympathetic nerve system activity and correlates with the regulation of blood pressure and peripheral vascular tone. High-frequency (HF) power reflects sinus arrhythmia and breathing activity and is strongly based on parasympathetic nerve system activity [20]. The ratio of LF to HF power is commonly interpreted as an indicator of the relative balance between sympathetic and parasympathetic activity, with higher ratios corresponding to higher relative sympathetic activity [21]. The total power is not a specific indicator of a particular type of autonomic nervous system expression, but is rather an indicator of the total amount of variability, which includes all of the different possible influences on heart rate variation [20].

CHHF may lower quality of life by interfering with daily life and restricting activities in the cold environment. Therefore, improving quality of life is another important goal in the treatment of CHHF. The SF-36 is a generic instrument to measure health-related quality of life and is widely used to survey physical and emotional health. The validated Korean version of SF-36 that will be used in this study was provided by the Health Assessment Laboratory (Boston, MA, USA). It consists of 36 questions grouped into eight dimensions: physical function (10 items), role limitations owing to physical health problems (4 items), bodily pain (2 items), general health perception (6 items), energy and vitality (4 items), social function (2 items), role limitations owing to emotional problems (3 items) and mental health (5 items). The number of questions for each health concept ranges from 2 to 10, and the number of response options per question is either two (yes or no) or six (none, very mild, mild, moderate, severe or very severe). Each of the dimension scores are expressed as a value between 0 and 100, with higher scores representing better health status [22].

### Safety assessment

All participants will be asked to record any adverse events in a diary during the administration and the follow-up period. All adverse events will be described in the case report form (CRF). The complete blood count (CBC), erythrocyte sedimentation rate (ESR), liver function test and renal function test will be assessed to determine the safety of the treatments at the completion of KRG or placebo administration. These analyses will be performed at an accredited laboratory. All values detected in the laboratory will be recorded by the investigator in the CRF.

### Sample size

This trial has a pilot characteristic to evaluate the efficacy of KRG on CHHF by using infrared thermography. Although there was no previous study that is the same in design, we calculated the sample size on the basis of a study that used similar variables. The previous study measured the tolerance time to cold stress to assess the efficacy of KRG on CHHF using a two-sided test, yielding a 5% significance level [16]. The formula for estimating the sample size is as follows:

$$n_{t}=n_{c}=\left\{{\left(Z_{\alpha /2}+Z_{\beta }\right)}^{2}\sigma^{2}\left(\lambda +1\right)/\lambda \right\}/{\left(\mu_{t}-\mu_{c}\right)}^{2}$$

In the previous study, the tolerance time to cold stress prolonged 0.35 minute in the KRG group compared with the control group (μ_t_-μ_c_), and a mean standard deviation (σ) was 0.84. In our study, the ratio (λ) of KRG to placebo is 1:1. With an 80% power (1-β) and a 5% significance level (α), assuming μ_t_-μ_c_ = 0.35 and σ = 0.84, a sample size of *n*_*t*_ *= n*_*c*_ = 32 patients per group is needed (*n*_*t*_, number of patients in KRG group; *n*_*c*_, number of patients in placebo group). Anticipating a drop-out rate of approximately 20%, the total sample size should be more than 80 women.

### Randomization

Randomization will be performed by an independent statistician by generating allocation numbers using a randomization allocation program. The participants will not be stratified. They will randomly be assigned into KRG and placebo groups at a 1:1 ratio. The investigator will be subsequently notified of the number assigned to each participant and the participants will be given a random number at their second visit. The allocation table of participants will be kept by an independent statistician until the end of the study.

### Blinding

The patients will be blinded to the treatment that they are provided. The investigator and clinical pharmacist will be also blinded to the randomization. Only the independent statistician will be aware of the randomization.

### Ethical approval

The protocol of this trial has been approved by the institutional review board (IRB) and the ethics committee of Kyung Hee University Hospital at Gangdong, Seoul, Korea. The permission number of this study is KHNMC-OH-IRB 2012–004 and the protocol identification number on ClinicalTrials.gov is NCT01664156. Written informed consent will be obtained from all participants prior to enrollment, and patients will be given adequate time to declare if they wish to participate before signing the consent form.

### Recruitment

Information will be sent by short message service (SMS) to patients with CHHF who were previously identified at the Kyung Hee University Hospital at Gangdong. Public advertisements will be placed in the newspaper and homepage of the hospital to recruit participants. Posters, brochures and banners will be placed inside the hospital.

### Concomitant therapy

During the clinical trial, patients will be prohibited from taking any kind of drugs or therapies that might affect symptoms related to CHHF. These include herbal medicines, acupuncture, moxibustion, cupping and infrared treatment. Furthermore, the patients will be prohibited from taking hypertensive or diabetic medications and thrombolytic agents that affect vascular or endothelial functions. However, the patients will be permitted to take medications that would not affect CHHF. For example, medications for a common cold, stomachache, diarrhea and menstrual pain will be allowed.

### Statistical analysis

An independent statistician will perform the statistical analyses of all data in a blinded manner. An efficacy analysis will be performed for both ITT (intention-to-treat) and PP (per-protocol) data set. Baseline characteristics such as age and body mass index (BMI) between the two groups will be compared using independent *t*-test or chi square test. The target variables for analysis in this study include the following: (1) change of skin temperature and VAS scores of cold hypersensitivity in the hands; (2) change of skin temperature and VAS scores of cold hypersensitivity in the feet; (3) RR of the skin temperature by the CST of the hands; (4) the DDD of the hands; (5) power variables of HRV; and (6) the SF-36. All values will be presented as mean ± SD. The paired *t*-test will be used to compare data between the baseline (visit 2) and after the treatment (visit 4) in each group. Comparison between the two groups will be performed using independent *t*-test at each time. If there are significant differences between the two groups at the baseline, we will compare the variables using ANCOVA. All statistical analyses of data will be performed by using SPSS version 17.0 (SPSS Inc., Chicago, IL), with *P*- values <0.05 representing statistical significance.

## Discussion

CHHF cannot be dissociated from Raynaud’s phenomenon (RP). A diagnosis of RP can be made when color changes occur in the skin of the digits of the hands and feet in response to cold temperatures or emotional stress [23]. RP is known to be caused by a transient cession of blood flow to the digits of the hands or feet [24] and is associated with various diseases, such as rheumatoid arthritis, scleroderma, dermatomyositis, hypertension and migraine [25]. However, RP can occur in the absence of these disorders, which is referred to as primary RP [26]. Although the pathophysiology of primary RP remains poorly understood, increases in sympathetic receptor activity in blood vessels, endothelial dysfunction and some central thermoregulatory defects have been suggested to contribute to the development of primary RP [24].

A survey was conducted regarding the prevalence of RP among 1,008 outpatients who complained of abnormal sensory symptoms in the hands and feet. The most common abnormal sensory symptoms, in order of descending frequency, were tingling, coldness and pain. Of the 510 patients (52%) who answered “yes” to the question, “Are your extremities unusually sensitive to cold?”, 290 patients (43%) showed RP [27]. These results suggest that CHHF, a conventional circulatory disorder, may be regarded as latent RP. Thus, it can be said that CHHF is RP without color changes of the hands and feet.

Infrared thermography, which has been used as a non-invasive investigative tool in medicine since the 1960s, can easily assess local differences in skin temperature. Infrared thermography is an easily applicable, well-established imaging method that can be used to create temperature maps of the skin with satisfying reproducibility [28,29]. Since cutaneous temperature depends on local blood perfusion and thermal tissue properties, infrared imaging provides important indirect information concerning circulation, thermal properties, and the thermoregulatory function of cutaneous tissues [30]. The subjective estimation of finger temperature has been shown to be closely related to finger temperature as measured by infrared thermography [31].

For the diagnosis of cold hypersensitivity in the hands, thermographic measurements will be performed at two paired areas (the palm and upper arm). A diagnostic cut-off value for cold hypersensitivity in the hands was determined in a previous study. When the criterion for the thermal difference between the palm and the upper arm was set as greater than 0.3°C, the sensitivity and the specificity of the test were 94.0% and 90.0%, respectively [4]. In the current study, we will diagnose CHHF according to this cut-off value.

The CST is applied to exposed skin areas with impaired thermoregulation [32]. The CST reportedly has high sensitivity and specificity for RP in comparison with healthy control subjects [33]. In addition, the CST was shown to have good sensitivity, specificity, and positive and negative predictive values for the diagnosis of digital vasospasm in patients with hand-arm vibration syndrome [34]. A previous study demonstrated a significant negative correlation between the RR of finger temperature after cold stress and VAS scores [35]. Therefore, RR after cold stress would be a good indicator for evaluating CHHF.

Among other methods, the measurement of skin temperature with the CST has been reported to be a good indicator of sympathetic responses [36], and has been used to evaluate central autonomic and peripheral vascular responses [37]. Cold stimulation increases sympathetic activity and the removal of the extremities from a cool environment results in prompt rewarming in the healthy subjects, with an RR of over 80% at five minutes. Therefore, a reduced RR indicates exaggerated sympathetic nerve function. According to previous works, a RR of <80% is considered clinically meaningful and suggests increased sympathetic nerve activities [38].

The sympathetic nervous system plays a major role in the regulation of cutaneous blood flow [39]. Therefore, sympathetic dysregulation has been hypothesized to be a factor in the genesis of RP. The exaggerated digital vasoconstrictor responses to cold, emotional stress, and postural changes [40] and the efficacy of both local anesthesia and sympathetic blockade in controlling vasoconstriction of RP [41] are consistent with a hypothesis of sympathetic over-activity. In a clinical examination, HRV spectrum analysis revealed significantly higher LF values in the RP group compared with the control group [42]. Another trial revealed relatively more sympathetic nerve system expression and less parasympathetic expression in a slow rewarming group after cold exposure compared to a normal group. Lower values were observed for all parameters after cold weather training for 15 months, and this was particularly evident for sympathetic nerve system expression, as indicated by lower LF values [43]. In the current study, we will evaluate autonomic nervous activity after the administration of KRG by using HRV.

Controversy exists in the literature regarding the effect of ginseng on autonomic nervous activity. Wild ginseng pharmacopuncture in healthy adults tended to activate the autonomic nervous system, particularly the sympathetic nervous system [44]. Another clinical trial showed higher parasympathetic and lower sympathetic activity modulation after the administration of Kampo including ginseng [45]. However, the investigators used wild ginseng pharmacopuncture or Kampo (a mixture of ginseng, oriental bezoar and glycyrrhiza), not ginseng alone. The current study is the first trial to evaluate autonomic nervous activity through the oral administration of KRG.

The DDD was shown to be a valuable parameter in discriminating between patients with primary RP and secondary RP due to systemic sclerosis. Previously, a DDD >1°C in any finger at room temperature was considered a specific indicator of underlying structural vascular disease [19]. Because the DDD is used as a parameter to evaluate the severity of vasoconstriction, the severity of CHHF can be measured by the DDD.

Although CHHF is poorly defined medically, complaints of CHHF are common in Asian women. Nevertheless, few studies have been conducted regarding the diagnosis and treatment of CHHF. The current study is the first trial to evaluate the efficacy of KRG on CHHF by using infrared thermography. We anticipate that this trial will provide basic evidence regarding CHHF and that the protocol for this trial will be a reference for designing further clinical trials.

## Trial status

The study was designed in 2012 and the first participant was randomized on 15 October 2012. Recruitment for this study is ongoing.

## Abbreviations

ANCOVA: Analysis of covariance; CBC: Complete blood count; CHHF: Cold hypersensitivity on hands and feet; CRF: Case report form; CST: Cold stress test; DDD: Distal-dorsal difference; ESR: Erythrocyte sedimentation rate; HRV: Heart rate variability; IRB: Institutional review boards; ITT: Intention-to-treat; KRG: Korean red ginseng; LF: Low frequency; PP: Per-protocol; RP: Raynaud’s phenomenon; RR: Recovery rate; SD: Standard deviation; SF-36: The 36-item short form health survey; VAS: Visual analogue scale.

## Competing interests

The authors declare that they have no competing interests.

## Authors’ contributions

KSP, JWK, JYJ and JML contributed to the funding and the design of the study. SJH, NRY, DSH, CHL, JBJ and KSL participated in the design of the study. IKY calculated the sample size and determined the methods of statistical analysis. KSP and JML prepared the manuscript. All authors read and approved the manuscript.
